# Provisioning, Authentication and Secure Communications for IoT Devices on FIWARE

**DOI:** 10.3390/s21175898

**Published:** 2021-09-02

**Authors:** Patrícia R. Sousa, Luís Magalhães, João S. Resende, Rolando Martins, Luís Antunes

**Affiliations:** 1 Department of Computer Science, Faculty of Sciences, University of Porto, 4169-007 Porto, Portugal; up201606761@fc.up.pt (L.M.); jresende@dcc.fc.up.pt (J.S.R.); rmartins@dcc.fc.up.pt (R.M.); lfa@dcc.fc.up.pt (L.A.); 2 Institute for Systems and Computer Engineering, Technology and Science (INESC-TEC), 4200-465 Porto, Portugal

**Keywords:** Internet of Things, FIWARE, authentication, secure communications, smart cities

## Abstract

The increasing pervasiveness of the Internet of Things is resulting in a steady increase of cyberattacks in all of its facets. One of the most predominant attack vectors is related to its identity management, as it grants the ability to impersonate and circumvent current trust mechanisms. Given that identity is paramount to every security mechanism, such as authentication and access control, any vulnerable identity management mechanism undermines any attempt to build secure systems. While digital certificates are one of the most prevalent ways to establish identity and perform authentication, their provision at scale remains open. This provisioning process is usually an arduous task that encompasses device configuration, including identity and key provisioning. Human configuration errors are often the source of many security and privacy issues, so this task should be semi-autonomous to minimize erroneous configurations during this process. In this paper, we propose an identity management (IdM) and authentication method called YubiAuthIoT. The overall provisioning has an average runtime of 1137.8 ms ±65.11+δ. We integrate this method with the FIWARE platform, as a way to provision and authenticate IoT devices.

## 1. Introduction

The Internet of Things (IoT) allows everyday objects (equipped with computational and communicative capacity) to connect to the Internet. The “things” can exchange data with each other and the Internet, making decisions automatically, even without human interaction. The IoT applications require platforms to facilitate its development process, including data transmission between heterogeneous devices with varying capabilities and different communication protocols. The literature presents several middleware platforms that serve as the underlying infrastructure for the development of IoT applications [1,2].

Smart cities use technology solutions to improve city services and residents’ living experiences. Sensors, networks, and applications collect relevant data, such as traffic congestion, energy usage, and air quality, to improve city services [3]. Cloud-based IoT applications receive, analyze and manage data in real-time to help municipalities, businesses, and citizens make decisions that improve the quality of life. The collection of traffic data to change traffic lights automatically is an example of these smart cities initiatives. Thus, some devices can collect traffic data, and when faced with congestion scenarios, the city can automatically manage the green light for longer, causing the traffic jam to decrease. Citizens also interact with smart city ecosystems using smartphones and mobile devices. Pairing devices and data with cities’ physical infrastructure and services can reduce costs and improve sustainability. Communities can improve energy distribution, optimize garbage collection, reduce traffic congestion and even improve air quality with the help of IoT.

The conception of IoT has founded the smart cities, which support the city operations intelligently with minimal human interaction [4]. However, the realistic implementation of smart cities is challenged on all costs of design and operation, heterogeneity among devices, enormous data collection and analysis, information security, and sustainability.

In this context, the European Commission made available the FIWARE platform [5], which is an open-source middleware developed to contribute to the creation of technologies aimed at the Internet of the Future and capable of meeting a wide range of requirements relevant for IoT platforms. There is a specific variant for IoT called FIWARE IoT stack. Its purpose is to enable devices to send and receive data through specific APIs to send data from the device to the cloud and receive commands. Other available API functions are described in the official stack document [6].

On these smart city platforms, one of the most important characteristics to consider is security and privacy [7,8,9]. With the growth of these technologies, there are more sensible data exchanged between devices and users. Furthermore, there are many security problems in smart cities [10,11] that can lead to catastrophic consequences. For example, suppose an attacker has access to a traffic light. In that case, it can give wrong information (leading to road accidents), or in a case more related to privacy, video surveillance systems can be hacked, and unauthorized users can access confidential images, exposing personal data. In terms of security, if these systems are hacked or fail, it can lead to catastrophic consequences.

### 1.1. Problem Statement

The security of IoT devices is an essential part of building these connections and interactions on platforms. Customers need to be confident that their data will not be shared with unknown entities, or tampered with [12], or even that their devices do unwanted operations. Authentication is an indispensable process, and generally, IoT platforms ask the customer to go through a manual process of provisioning the IoT device through the generation of shared keys that ensure the functioning and secure communication of the device. Some devices are already ready to be provisioned with some custom platforms semi-automatically, but not all of them can provide secure provisioning.

Most authentication solutions available on these platforms (FIWARE, for example) [13,14], namely KeyRock or KeyStone, are more focused on user authentication. Devices can also authenticate, either with OAuth2 mechanisms or with shared key mechanisms between devices. However, the OAuth2-based mechanism is more user-friendly for human/users authentication, as the implementation requires traditional username/password registration. Furthermore, impersonation attacks can be performed if an attacker discovers the shared key and the device ID.

Furthermore, there is an obstacle towards the development of solutions based on the Trusted Platform Module (TPM) to accomplish a chain of trust from hardware to software components. Thus, past work [14] open up a future research challenge focused on a USB security token-based mechanism as a possible solution. Given the aforementioned limitations and challenges, can we devise a system with secure tokens to provide several benefits, namely device identity, authentication, end-to-end communications, offline cryptographic assets, and resistance to MiTM?

In this paper, we explore an authentication mechanism for IoT devices integrated on FIWARE. The paper explores the lack of secure provisioning, authentication, and communication mechanisms between devices independent of FIWARE, but that can be integrated into the platform so that devices that belong only to a trusted pool can be considered secure. The motivation for this work is related to the need for device authentication and identity, which must be considered a priority. In the context of smart cities, it is necessary to ensure the definition of a device identity to eliminate impersonation attacks, preventing one device from impersonating another and sending data with its identity.

Thus, it is also necessary to ensure that the communication between two devices is encrypted and securely transported over the communication channel to guarantee that the data sent by a device is the same that arrives at the destination device (without tampering, maintaining integrity). These mechanisms can eliminate the possibility of eavesdropping on information by a malicious or passive party, maintaining privacy, and eliminating the possibility of Man-in-the-Middle (MitM) attacks.

This document proposes an IoT device provisioning mechanism that provides identity, authentication, and secure communications using a secure token to provision IoT devices and their integration into the FIWARE platform. It is possible to have device pools, which in addition to authenticating multiple devices, can also authenticate with each other (for example, different entities or departments), allowing you to scale the solution to provision and authenticate multiple devices without the need to authenticate across all of them (1 to 1).

### 1.2. Contributions

- Device Identity: A solution that relies on the combination of a secure token (capable of generating OTP and storage of a PKI) with cryptographic algorithms to provide an identity to devices. Managers can authenticate the trusted devices in their pool, giving them an identity;
- Devices’ Pools: After device provisioning, the system can provide a decentralized architecture where the trusted devices can communicate end-to-end between each other (if they are in the same or trusted pools). Different pools can be trusted between each other if both managers agree on that (for this reason, we consider that the scalability is better than other systems that need to authenticate all devices between each other);
- Offline Cryptographic Assets: This system’s main advantage is the use of ephemeral keys for clients and the manager’s offline cryptographic assets (with the usage of the secure token), including storing the private key isolated from network access and kept in a powered-down state. Even if the secure token is stolen or lost, it needs a PIN (Personal Identification Number) to access the keys. As the secure token stores all the cryptographic keys, it eliminates the hassle of having all of the cryptographic assets on the managing device, which would lead to a single point of failure (SPOF).
- YubiAuthIoT Full Implementation: We have a fully working implementation of the YubiAuthIoT method. Section 4 has the setup description as an independent authentication method. We describe all the implementation processes, including the configurations of the YubiKey cryptographic algorithm, local certificate authority, OTP Server, and the discovery process. Then, we provide some performance results.
- FIWARE Integration and Full Implementation: Section 5 and Section 6 describe the integration with the FIWARE, which involves many components that must be deployed to achieve the desired functionality and security. We describe all the components that compose the implementation with FIWARE, including the communication between nodes, connection with AuthzForce for authorization policies, and connection with Orion. We chose integration with FIWARE to test the integration, but the method YubiAuthIoT can be integrated on any other platform, as the implementation is independent (described in Section 4).

### 1.3. Outline

The paper is structured as follows: Section 2 describes the related work solutions for identity management and authentication across multiple IoT scenarios. Section 3 presents the Identity Management and Authentication method that we created for securing the communications end-to-end between IoT devices, describing the architecture. Section 4 shows the evaluation of the YubiAuthIoT method, namely the Setup, Implementation, and performance results. Then, Section 5 shows the integration between our method (YubiAuthIoT) with FIWARE, describing architectural and implementation details. Section 6 describes the YubiAuthIoT and FIWARE integration deployment, namely the setup and implementation details. Section 7 describes the security analysis composed of a threat model and some attack scenarios. Finally, Section 8 gives an overview of the future research challenges, and Section 9 has the conclusions of the work.

## 2. Related Work

Many applications provide identity, authentication, and authorization across multiple contexts for IoT scenarios [15,16,17,18,19,20,21,22,23], and different authors present many taxonomies. Shubham Agrawal et al. [24] claim that there are different authentication schemes, named: OTP, Zero-Knowledge proof, Mutual Authentication, Public Key Cryptography, and Digital Signature. On the other hand, VL Shivraj et al. [25] refer to a different categorization: mutual authentication schemes, two-party authentication through a trusted party with key exchange, session key-based authentication, group authentication, directed path-based authentication scheme, OTP, and SecureID authentication schemes. Nidal Aboudagga et al. [26] have presented a document with a taxonomy and research issues in the authentication protocols for ad hoc networks.

PKI provides important core authentication technologies for IoT. PKI creates digital certificates that map public keys to entities that securely store these certificates in a central repository and revokes them if needed. Any device can verify the integrity and ownership of a public key in this type of infrastructure. Previous studies [27] show that 42% of devices will continue to use digital certificates for authentication and identification in the next two years. The SSL/TLS [28] or Kerberos [29] are some examples of authentication systems based on a PKI.

Regarding FIWARE, KeyRock [14] is the component responsible for Identity Management. Using KeyRock (with other security components such as PEP Proxy and Authzforce) enables users and devices to communicate with OAuth2-based authentication and authorization. However, although IoT devices can authenticate with FIWARE with these methods, it is not done for that purpose, as it uses the traditional username/password registration. Even if this type of system is not the most advanced implementation in terms of security and usability, it can still be a good mechanism because it integrates seamlessly with the FIWARE architecture but is more user-oriented. KeyRock also requires a SQL database to store the encrypted user credentials.

Luciano Barreto et al. [13] present an overview of some IoT cloud-focused device authentication solutions and assert that current security technologies often do not fully include the tools needed to handle such a scenario. The authors propose that even considering the KeyRock [14] and Keystone [30] solutions on FIWARE technology, there is an obstacle towards the development of solutions based on Trusted Platform Module (TPM) to accomplish a chain of trust from hardware to software components. Thus, the authors open up a future research challenge focused on a USB security token as a possible solution-based mechanism, which is the focus of our solution in this paper.

For this reason, it is interesting to consider other solutions for the authentication of IoT devices in FIWARE.

Secure Provisioning for Achieving End-to-End Secure Communications [31] provides a concept for provisioning IoT devices that adopts an architecture where another device acts as a manager that represents a CA, allowing it to be switched on/off during the provisioning phase to reduce SPOF problems. The solution combines One Time Password (OTP) on a secure token and cryptographic algorithms on a hybrid authentication system. In this solution, the certificates are signed on the manager device, having a SPOF because it stores all the keys on the manager device. Therefore, the solution does not explore the functionality of Public Key Cryptography Standards (PKCS)#11 on YubiKeys to create and manipulate cryptographic tokens. Thus, we use a solution in this paper based on that, but we use the YubiKey to perform all the cryptographic operations inside it, ensuring that the private key never leaves the device to avoid the possibility of private key theft in a possible network intrusion. Furthermore, we change the cipher algorithms. The authors use an Elliptic Curve Integrated Encryption Scheme (ECIES) for authentication of the receiver. Users can encrypt an ephemeral key using the data key and then send it, and if the other party can decrypt, users authenticate the recipient. However, in this case, both parties established an ephemeral data key to encrypt an ephemeral authentication key. It would be much easier to use the data key for authentication. For this reason, in this paper, we choose the Elliptic Curve Digital Signature Algorithm (ECDSA) for signing and verification and Elliptic Curve Diffie–Hellman Key Exchange (ECDH) for encryption.

## 3. IoT Devices IdM and Authentication

In this section, we will present a solution (YubiAuthIoT) based on the concept provided by Patrícia R. Sousa et al. [31], but with some improvements that ensure device identity, scalability, offline cryptographic assets, and resistance to MitM.

YubiAuthIoT presents a novel approach for provisioning IoT devices that combines public-key cryptography algorithms with OTP inside a secure token. The secure token acts as offline storage for private keys, allowing access to cryptographic operations to be kept offline without access to the network, contrary to the original solution. Our solution uses a manager device that acts as an OTP Server that can be switch off during the provisioning phase to reduce a SPOF problem. This way, device identity is guaranteed by physical access to this physical token.

### 3.1. Manager Setup Phase

The Certificate Authority (CA) subsystem is a combination of a secure token and a manager device. The secure token plays an essential role in a certification system, supporting the combination of OTP with PKCS#11 to create and manipulate cryptographic tokens (The PKCS#11 standard/protocol is widely used by applications that use cryptographic operations with non-exportable keys, as the protocol defines a standardized specification for interaction with cryptographic hardware (Smartcards, Tokens and Hardware Security Module (HSM)). It is an abstract layer to perform the separation of the keys from the operations, allowing them to perform operations on cryptographic objects, such as private keys, without requiring access to the objects.). In our system, we used a secure token to perform all the cryptographic operations inside it, ensuring that the private key never leaves the device to avoid the possibility of private key theft in a possible network intrusion. This device must be reliable and controlled only by trusted people, such as the network owner. All certificates signed by the device will be implicitly trusted. The systems that manage Public Key Infrastructure (PKI) require a high-security degree and are on an isolated machine.

As the secure token stores all the cryptographic keys, it eliminates the hassle of having all of the cryptographic assets on the managing device, which would lead to a SPOF. On the other hand, the device manager has an OTP server that allows authentication with a secure token (“something you have”), proving that the device trying to authenticate has the secure token. For this reason, anyone who has access to this secure token can authenticate with the manager. The OTP server can be switched off when not in use, avoiding possible SPOF.

### 3.2. Device Authentication

**Figure 1:**
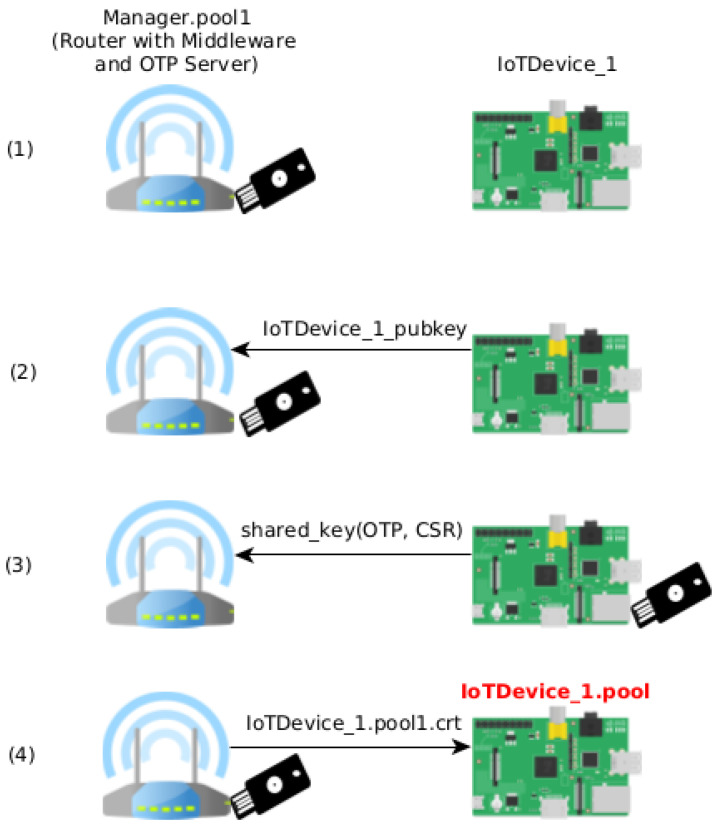
Setup and authentication.

The authentication between a new device and the manager is needed to add new devices to the trusted device pool. We use a combination of public-key cryptography algorithms with OTP to authenticate a new device. In practical terms, the network owner inserts the secure token into the target device to add it to the trusted device pool. Figure 1 shows the whole process.

This section describes the cryptographic algorithm used in this proposal and then provides a detailed description of the entire process of provisioning new devices.

#### 3.2.1. Cryptographic Algorithms

As cryptographic algorithms, we choose ECDSA for signing and verification and ECDH for encryption.

We choose Elliptic-Curve Cryptography (ECC) because it is better for low-resource devices, as ECC requires fewer resources and provides the same security level as Rivest–Shamir–Adleman (RSA) cryptography with a smaller key [32].

ECDH is a shared-secret derivation protocol that uses the elliptic curve form of the Diffie–Hellman (DH) protocol. In this protocol, two parties can agree on a shared secret over an insecure channel using the knowledge of their own “private key” and their partner’s “public key” to generate a shared secret. Generally, the private keys are random numbers used for the key negotiation and then discarded (ephemeral). According to the NIST SP 800-56A [33], there are three key agreement categories: static–static, static–ephemeral and ephemeral–ephemeral. On the static–static scheme, there are no ephemeral keys on usage; on static–ephemeral, it only generates one ephemeral key pair for one of the parties; and finally, on ephemeral–ephemeral, each party generates an ephemeral key.

In general, a static key remains the same over a long period. However, an ephemeral key has a very short lifetime and is recreated for each session. The “static–static” scheme does not provide forward secrecy, which means that if an adversary finds either one of the private keys, then the shared secret can be calculated (using the other party’s public key), and all security is lost. The “ephemeral–ephemeral” scheme provides forward secrecy, which means that past sessions are still secure even if an attacker finds one or both private keys, as this scheme generates a new key pair for each key agreement. It is not accurate stating the ECDH does not provide authentication while using ephemeral keys, although it is required to authenticate the public key’s exchange. However, the protocol can use ECDSA—the elliptic curve form of DSA, to solve the lack of authentication. DSA authenticates digital content because a valid digital signature gives a recipient reason to believe that the message was created by a known sender, such that the sender cannot deny having sent the message (authentication and non-repudiation) and that the message is the same—without changes (integrity). This authentication includes the key agreement parameters used to derive the master secret and includes the session key’s correctness. In brief, it can authenticate the handshake of the TLS protocol so that it can provide authentication for ECDH [34].

Finally, the “ephemeral–static” scheme does not provide forwarding secrecy because if an adversary finds the static private key, the shared secret can be discovered, as it can be calculated. To the best of our knowledge, there are no hardware tokens with ephemeral keys. Our system does not require “forward secrecy” property for the manager because the secure token keeps keys offline, and an attacker needs to have physical access to the physical token and know the PIN to access the private key. For this reason, we chose the “ephemeral–static” scheme.

#### 3.2.2. Middleware’s Authentication Process

The authentication process is depicted in Figure 1 and shows its four distinct phases.

After the manager sets up the OTP Server, the manager is ready to receive new authentications from IoT devices (1). A new device can send its ephemeral public key to the manager to initiate the conversation (2). As the key is ephemeral, the device needs to authenticate itself with the manager. To achieve this goal, it uses the manager’s public key (stored on the secure token) and its ephemeral private key (correspondent to the ephemeral public key sent on the first step) to derive the ECDH shared key. Then, the device sends a Certificate Signing Request (CSR) generated with the ECDSA private key and an OTP generated with the secure token to the manager, both encrypted with the derived shared key (3). The server also derives an ECDH shared key with its private key and the device’s ephemeral public key received on the previous step. The manager can then decrypt the device’s communication that contains the OTP and CSR. If the OTP is considered valid by the OTP Server, he knows that the client is in the physical presence of the physical token. If this is the case, the manager signs the CSR, generating a signed x509 certificate, and sends it back to the client (4). It attests that the new device is now on the device’s trusted pool. These certificates establish trust among the client devices (without the intervention of the manager device).

The security token has a PIN to protect the signing action to ensure that no one, except the owner, uses that token to sign.

After this process, the shared key must be discarded (deleted) from the devices.

### 3.3. Decentralized Secure End-to-End Communications

After the discovery process, devices need to authenticate among themselves without intervention from the manager. For the mutual trust, both devices must exchange the manager’s signed certificates. When the device IoTDevice_1.pool1 wants to communicate with others, such as IoTDevice_2.pool1, they need to exchange their certificates to prove to each other that they are trusted.

As all the clients keep the manager’s public keys, the signatures of their certificates can be verified mutually. Then, both clients exchange ephemeral keys signed with ECDSA. With access to the certificate signed by the manager, they can extract the public key and verify the signature of the ephemeral public keys to prove that it is the same person who has them (and, therefore, is authenticated). After exchanging their public keys, each client can derive an ECDH shared secret to communicate (with their private key and the other’s public key).

The security of the transmission of the .crt is implicitly given by the possession of the private key that only the owner has access to, so the authentication is guaranteed (even if someone eavesdrop on the channel and stole the .crt, they do not have the private key associated to it).

### 3.4. Merge Two Trusted Devices Pools

**Figure 2:**
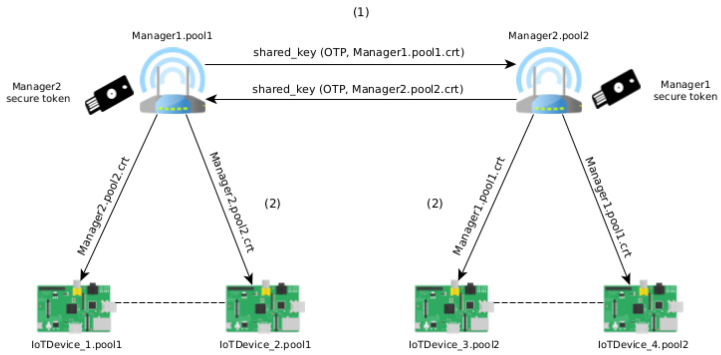
Merge two trusted pools.

One of the options of this system is the possibility of authenticating pools between them. This option has the advantage of authenticating devices from different pools (for example, in the context of smart cities, between different departments) so that the devices do not need to authenticate between all (1-to-1). For that, if two managers want to merge their pools, they need to authenticate with each other—step 1 in the Figure 2. After this, both pools have their devices authenticated with each other. This option ensures that authentication between devices is the responsibility of managers.

Then, each device updates its list of trusted devices with the certificates of the devices of the other trusted pool—step 2 in the Figure 2. Thus, all devices can verify which devices are trusted. Likewise, and in the presence of a misbehaving device, it is possible to revoke its certificate, and therefore, revoke the trust between devices. Here, the Certificate Revocation List (CRL), which stores the information about the provisioning devices that currently have their certificate revoked, is updated. Only the manager can revoke devices of the pool managed by it. This list is shared between pools when there is authentication between them.

Figure 2 represents the merge process between two pools. Authentication between managers is done through the same process described in Section 3.2. Then, the information (updated trusted certificates) is shared between all devices in the different pools.

## 4. YubiAuthIoT Evaluation

This section describes the setup phase and the implementation details regarding the YubiKey cryptographic algorithms configuration, the local certificate authority setup, the discovery process, and the setup of the OTP Server. Then, finally, we describe different results regarding the provisioning phase, mainly the sending and encryption execution time of the OTP verification and public key.

### 4.1. Setup

For implementing a Proof-of-Concept of YubiAuthIoT solution, we use three Raspberry PI 3 (RP3) Model B+ and a YubiKey NEO. We use Raspberry PI because they are commonly used in IoT, like in some smart cities deployments [7,35,36,37,38]. In these deployments, Raspberry PIs are used as processing units in Data Collection Units (DCU), together with sensors and a control board to interface the processing unit and the sensors.

The devices connect to an UniFi AC Pro AP to mimic a WiFi deployment. The YubiKey NEO represents the secure token. One RP3 works as the manager (that acts as a router) and the other two as clients, with all the RP3 having the Raspbian operating system.

As the OTP Server can represent a SPOF, we created it as a service that can be switched off when not used to avoid the possibility of centralized attacks.

We assume that the RP3 connects by power and does not use extra batteries, such as power banks. For measuring the energy consumption, we use a direct plug-in power meter from efergy.

### 4.2. Implementation

This section describes the implementation of YubiKey Cryptographic Algorithms Configuration, Local Certificate Authority, Discovery Process, and OTP Server.

#### 4.2.1. YubiKey Cryptographic Algorithms Configuration

Regarding the YubiKey cryptographic algorithms configuration, a YubiKey allows us to generate OTP and supports Personal Identity Verification (PIV) [39] card interface. PIV enables RSA or ECC sign/decrypt operations using a private key stored on a secure token, such as smart cards, through the *PKCS#11* engine. *PKCS#11* bridges the gap between OpenSSL and YubiKey. A PIV-enabled YubiKey contains different slots capable of holding an X.509 certificate and the accompanying private key.

For the authentication process, we use Python 3.2, OpenSSL, the OpenSSL *PKCS#11* engine from *OpenSC*, the *p11tool* from *GnuTLS*, and the Yubico PIV tool for interacting with the PIV application on a YubiKey. With these tools, we can build a CA generated inside the YubiKey through the *PKCS#11* support.

This work uses a YubiKey for the OTP generation and stores the private keys for signing and encryption. We focus on two YubiKeys’ slots, mainly: 9c (for digital signatures) and 9d (key management). In a nutshell, we will use slot 9c for certificate signing purposes, and we are going to use slot 9d for encryption for confidentiality purposes, therefore, to decrypt content using the private key stored on YubiKey. Both slots require a PIN to perform operations with the private key. On the 9c slot, we create a 256-bit ECC key pair to use a 256-bit ECDSA. On the 9d slot, we also create a 256-bit ECC key pair for encryption and decryption. If the slot holds the EC key, we will perform ECDH and return the shared secret.

#### 4.2.2. Local Certificate Authority

For setting up the certificate authority, it is necessary to have some files located in a folder to store the certificates generated, and there are some main files and folders for that. The certificates are stored in a *certs/* directory and are listed in the *index.txt* file. The first field describes the certificate status, i.e., V for valid certificates and R for revoked certificates, the second is the issuing date, the third has the certificate serial, and finally, the last one contains the certificate name (Organization and Common Name). The manager device stores all this information.

#### 4.2.3. Discovery Process

After successful authentication, the device now belongs to a new domain. Then, we need to ensure that other services or entities can discover the new device inside the network.

We chose to use Zeroconf [40] to support the entire discovery process within our solution. It is a commonly used technology with wide commercial adoption by companies, such as Apple, which uses *mDNS* to locate any connected speakers, Apple TV, and others.

So, on this configuration, the domain of devices is changed from *IoTDevice_1* (for example) to *IoTDevice_1.pool1* (Example on Figure 1). When advertised on the network, the additional nodes can recognize them from the same domain. In the beginning, only the manager has their hostname along with the domain (pool1, chosen for this paper). However, all the trusted authentication devices must have the same domain when there are more authenticated devices. At this stage, devices can make a peer discovery to find devices with the same domain.

#### 4.2.4. OTP Server

For the OTP Server, we used an implementation of the YubiKey server based on Validation Protocol Version 2.0 from Yubico [41] and YubiKey-server repo from GitHub [42].

Basically, on this implementation, the server runs locally without the need for connecting to official servers. It stores all information inside an SQLite database.

We can enroll new keys on the system with the public key and the AES secret key. Then, it is possible to validate the OTP based on a query HTTP to the server. The response is composed of the OTP and Status (OK, REPLAYED_OTP or INVALID).

So, after the enrollment of the manager’s YubiKey, it is possible to authenticate with the manager with the YubiKey.

Devices attempting to authenticate run a standby service to read a public key from the *stdin* of the YubiKey (to encrypt the communications) and to perform the communication with the manager to send the OTP.

### 4.3. Results

During the device provisioning, there are some exchanges between the manager and the device being authenticated (Figure 1).

We do not measure the time required to create the sockets, but rather the time of sending the encrypted and decrypted data and, respectively, the process of encryption and decryption.

We collected ten-time samples from the provisioning phase for the manager and the device.

From the manager interactions, we have a mean runtime of 615.1 ms ±9.01, while in the client interactions, we have a mean runtime of 522.7 ms ±56.1. The overall provisioning has an average runtime of 1137.8 ms ±65.11+δ, where δ is the time required to insert the PIN when performing the cryptographic operations.

We calculate the verification’s runtime with *time.time()* on the authentication’s Python implementation, which returns the system date and time.

In terms of energy consumed, the RP3 switch on spends only 2.2 Wh without services running. When we did encryption and decryption, the energy spent was not significant, having just varied 0.4 Wh up and down.

The energy consumed by the device manager when the YubiKey-server is running and on the OTP validation process was 2.2 Wh, not varying more than 0.1 Wh up.

At the setup time, the manager makes the provisioning of their devices. Measuring the concurrent requests to the server is not our focus because it is an unusual situation. However, for the communications and the OTP validator, it is important to analyze the scalability. As far as communications are concerned, the scalability is proportional to that provided by the ECDH-ECDSA communication scheme.

Regarding the validation server, we present the scalability results given by the Locust tool [43]—a scalable performance testing tool. We test with 500 users (peak concurrency) at a spawn rate (users started/second) of 0.5. We get an average response time of 4.55 ms, where the minimum response time was 0.84 ms and maximum response time was 59.40 ms for an average content size of 147 and 83.86 requests per second.

**Figure 3:**
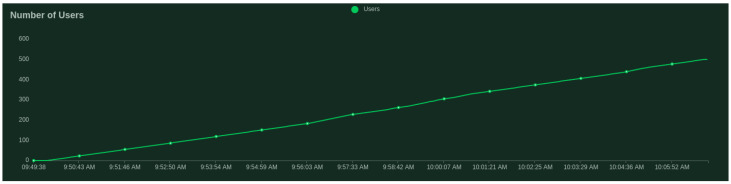
Number of users tested for the validation server.

**Figure 4:**
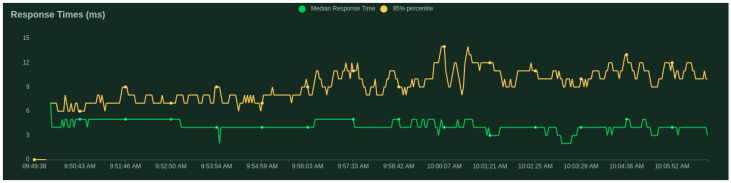
Response times (ms) per number of users.

Figure 3 and Figure 4 show that the median does not change significantly with the increase in clients.

## 5. Integration of YubiAuthIoT and FIWARE

FIWARE is a platform (or framework) that contains several open-source components that can be used together or individually and has third-party components to implement intelligent designs. FIWARE technologies aim to combine the Internet of Things with Context Information Management and Big Data services in the cloud to facilitate the processing, analysis, and visualization of this information. FIWARE can be used to implement well-designed Smart Cities, Smart Agriculture, and Smart Industry solutions.

FIWARE also has built-in components that allow connection to IoT devices, real-time event data processing, and Big Data analysis and incorporation of advanced Web User Interface features, such as Augmented Reality, 3D visualization, among others.

FIWARE has a set of APIs and software components for the rapid development of applications in contexts such as the Internet of Things (IoT); real-time data capture; context data management; data and big data analysis—requirements aligned with the basic needs for the development of applications for smart cities. It has two main objectives: to create the foundations for an open technology standard (FIWARE) and to create an open and technology-oriented innovation ecosystem. APIs are implemented through components called Generic Enablers (Generic Enablers, or GEs, are software tools offered by *FIWARE*) (GEs). *FIWARE* enablers [44] can be used to create an IoT platform, and *FIWARE* IoT Stack is based on them. There are different FIWARE components, and we can highlight them: IoTAgents (IoTA), Context Broker (Orion), and some Security Components, such as IdM, Policy Enforcement Point (PEP), and Access Control (AC).

**Figure 5:**
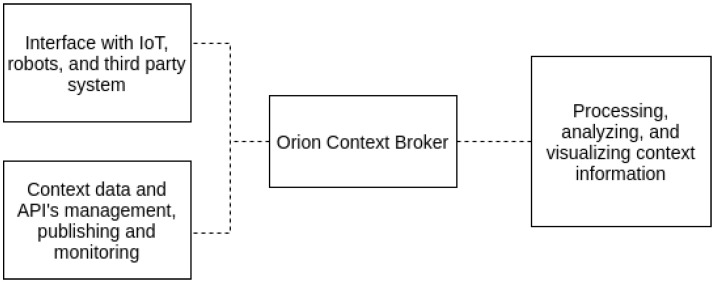
FIWARE Orion Context Broker representation.

The main component of FIWARE is the Orion Context Broker that provides the solution to manage, update and access context information. Using Orion Context Broker, it is possible to establish the link to other sets of complementary elements of the FIWARE (Figure 5): Interface with IoT, robots and third party systems; context and management data from APIs, publishing and monetization; and processing, analysis, and visualization of information from context.

Context Broker (Orion) allows us to model and gain access to context information independently of the source. Orion Context Broker is an implementation of the Publish/Subscribe Context Broker GE, providing an NGSI interface developed as part of the FIWARE platform. Orion allows managing the lifecycle of context information, including updates, queries, registration, and subscriptions. Context information consists of entities (e.g., a car) and their attributes (e.g., the speed or location of the car). Thus, Orion Context Broker can be connected to various IoT Devices, called Entities, which monitor environmental parameters such as Temperature, Brightness, and Acoustic Noise.

This section describes the integration between the YubiAuthIoT mechanism discussed in this paper and the FIWARE platform. Additionally, we provide a use case of both components on a smart city scenario, stating all the details that allow the establishment of secure and authentic communication channels between IoT devices and a smart city platform powered by FIWARE.

### 5.1. FIWARE Identity Management

**Figure 6:**
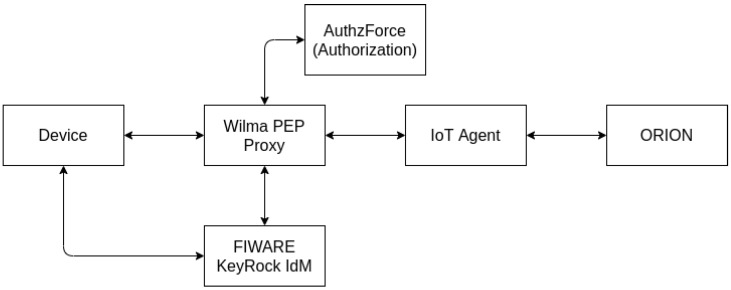
Simplified representation of the standard authentication FIWARE deployment.

The main components on the security of FIWARE [45] are IdM, Policy Decision Point (PDP), and Policy Enforcement Point (PEP) (Figure 6).

FIWARE Orion, the default implementation of the context broker GE, does not provide “native” authentication by default nor authorization mechanisms to enforce access control policies. Instead, it integrates with other FIWARE components such as FIWARE’s PEP Proxy [46] GEs, which provide the authentication and authorization capabilities to Orion.

Two PEP GE implementations can work with Orion Context Broker, namely Wilma [47] and Steelskin [48]. A PEP’s job is to be a proxy between the requests to Orion and the Orion itself, forwarding the communication to Orion if that matches the policies in place. Otherwise, the communications are discarded and return a forbidden error. A PEP does not have an Identity Management (IdM) for authentication management, policy creation, or decision capabilities. For a PEP to fulfill its role on the FIWARE architecture, it needs to connect with two additional FIWARE components: an IdM GE like KeyRock and a PDP GE such as AuthzForce [49]. The IdM is usually a server responsible for registering and authenticating users, being authentication performed via OAuth2 using access tokens placed on the communication HTTP headers. KeyRock provides a REST API and a web-based identity management interface to create users, roles, and permissions. The policy management, part of the authorization component of a security manager, is performed by the PDP GE, which provides mechanisms to create domains and policies, update them if needed, and also provides policy decision mechanisms, on which the user access request is validated according to the active policies returning an “Allow” or “Deny” decision. FIWARE’s Authzforce—the default implementation of the PDP GE, uses the eXtensible Access Control Markup Language (XACML) to build the policies and domains needed, and every policy decision request must be encoded in the same XACML format.

When all the components are available, the PEP GE checks the user authentication near the IdM GE, and if the authentication token is valid, this verification succeeds. After the successful verification of the authenticity of the user, PEP gathers information about the request, such as the entity ID that the user is trying to access, the HTTP method, and the request body, and sends it to the PDP, which returns a “Allow” or “Deny” decision. If either IdM or PDP returns an error or a deny result, the communication is not forwarded to Orion, protecting it from unauthorized access to information.

### 5.2. YubiAuthIoT and FIWARE

Without YubiAuthIoT, Figure 6 provides a simplified representation of the standard FIWARE deployment for authenticating devices.

**Figure 7:**
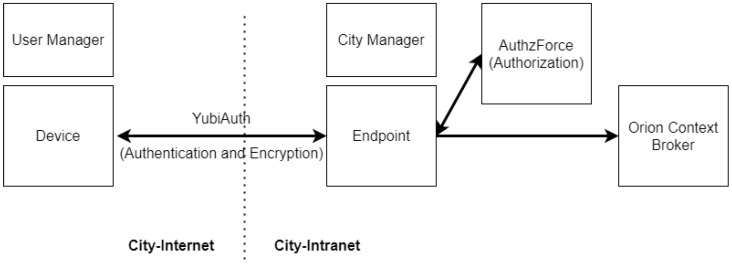
FIWARE integration (Orion Connection).

YubiAuthIoT seats on FIWARE architecture as an IdM, as it provides proofs of authentication and a secure channel provider. However, due to incompatibilities, it cannot be combined with existing PEP implementations. To integrate with FIWARE, we needed to develop an additional component that replaces the PEP on the FIWARE architecture, acting as a proxy between the requests to Orion and Orion itself. This new component aims to decrypt the incoming messages (as an IoTAgent), check the certificates for authentication, gather information about the request, make a policy decision request to a PDP, and if the result is favorable forward the message to Orion, completing its functions. This component is called Endpoint (Figure 7).

Note that YubiAuthIoT can be integrated into other systems than FIWARE. Here, we integrate with the Orion component to provide authentication and authorization, controlling requests and responses. However, YubiAuthIoT is independent and works as a provisioning method to authenticate devices, to provide end-to-end secure communications between trusted devices, totally independent from FIWARE.

**Figure 8:**
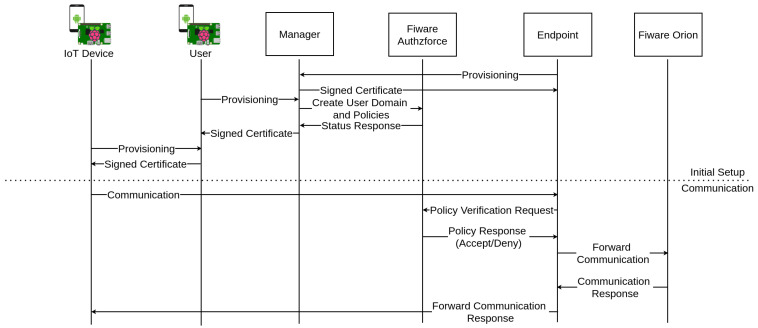
FIWARE authentication with connection to YubiAuthIoT.

Some cities have centralized services where users can access information about the city-state, such as weather information or real-time traffic. FIWARE Orion sits on a centralized infrastructure where it receives and distributes information. To adapt to a smart city infrastructure, we decided to make our PKI-based infrastructure tree-like, using a central city manager as our root CA and generating smaller sub-managers (subCAs) to manage an unlimited number of IoT devices. It is useful to remember that YubiAuthIoT does not require the manager nodes always on, so SPOF attacks are severely mitigated. Additionally, having multiple sub-managers, each one can manage a subset of devices, increasing the system’s reliability and usefulness. In a smart city, sub-managers can be companies or users with devices deployed to send data to the city and each other. Figure 7 aims to provide the general integration between the components that join YubiAuthIoT and FIWARE together, and Figure 8 shows the communication between the components in more detail.

As shown in Figure 7, the YubiAuthIoT component is responsible for making the authentication and encryption of communications between the IoT devices and the endpoint (controlled by the city manager). The endpoint is our custom PEP that uses the authentication information from YubiAuthIoT and information about the request to query the PDP (Authzforce) to decide the forwarding of messages to Orion. If the PDP result is favorable, the message is forwarded to Orion.

**Figure 9:**
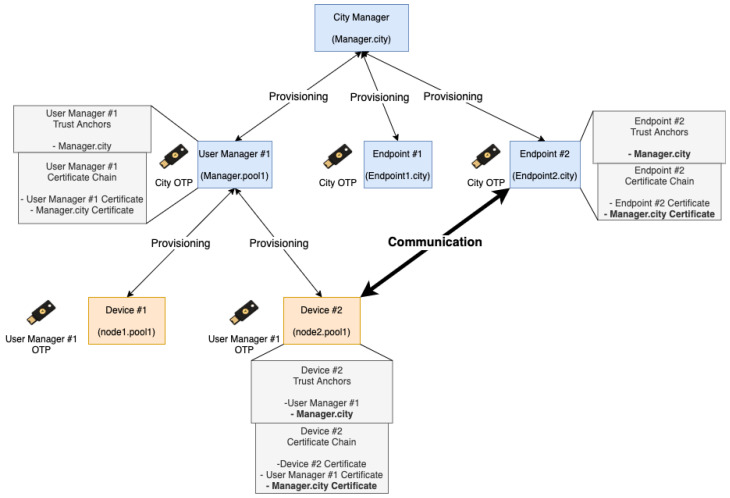
Smart cities use case.

Figure 8 represents the flows between the different components. Before any communication can be forwarded to Orion, the Endpoint device must be provisioned to ensure the authenticity of the endpoints when a IoT device wants to communicate with it. The City Manager (rootCA) is the device that provisions the Endpoint devices (can be more than one). The next device to be provisioned is the user/company manager (sub-CA) responsible for managing and authenticating their IoT devices’ subset. Then, the user/company manager provisions the IoT devices. It is important to note that every provision process occurs as detailed previously in Section 3. At this point, it is important to talk about trust anchors. The trust anchors are the certificates that are used on certification verification as the basis of trust. If the certification chain of a certificate contains some certificate that belongs to the trust anchors of a device, the certificate authenticity verification will succeed, and the certificate is trusted; otherwise, the certificate is untrusted. In the current implementation of YubiAuthIoTs tree-like PKI, the trust anchors of a node are all the parent nodes’ certificates until reaching the root on the PKI tree. Figure 9 shows the trust anchors placed on a smart city provisioning use case. The certificate chain is created by adding the device certificate on top of the parent certification chain. The certification chain of the rootCA is its certificate. Developing the certification chains and trust anchors give advantages regarding the communication with all the nodes on the PKI tree, including nodes belonging to other users/company managers and nodes belonging to the city. The FIWARE components described in this figure are placed internally within the smart city infrastructure and are only accessible by endpoint nodes and the city manager (rootCA), not being connected to the PKI tree generated with YubiAuthIoT. Restrictions to unmanaged device provisioning to the city, devices that have no user/company manager(sub-CA) associated can be easily provided as the physical security token is needed for the provisioning to succeed.

It is also possible to have user/company managers that are not connected to the smart city, making them the rootCA of their own devices, but when the manager connects to the city, it becomes an intermediate CA. With this, the user’s devices’ certificates will have the city in the certification chain and are authenticated to communicate with the endpoints for Orion. Figure 9 represents the devices’ provisioning process using YubiAuthIoT’s provisioning process. The construction of the trust anchors and certification chain is also shown.

After the provisioning between the manager and the endpoint in the provisioning phase, the manager sets up user domain and policies for the authorization grants on access to Orion. The policies are defined on the AuthzForce component that controls the access to Orion. We have two authorization policies: Each manager added to the city (intermediate CA) will force the creation of a domain whose name is the name of the manager’s pool (Manager.pool1 manages pool1), and each device that has the manager referred to above as an intermediate CA can only insert/edit/delete data within the same pool, and cannot edit data from other pools, meaning that editing data from other users is not possible. Then, the endpoint verifies the request information and certificate to grant or deny the access permission. As the policies installed in AuthzForce only allow the device to operate on entities within the same domain, attacks caused by other users are mitigated. The device can only communicate to the endpoint if the user who provisioned that device has provisioned in the city (the city is the root CA, the user is sub-CA). Otherwise, certificate verification fails on the endpoint device, and communication is canceled.

It is important to note that all the communication between devices within YubiAuthIoT is done using confidential, integral, and authentic channels with AES and digital signatures, using ephemeral symmetric keys and the certificates generated on the provisioning phase.

**Figure 10:**
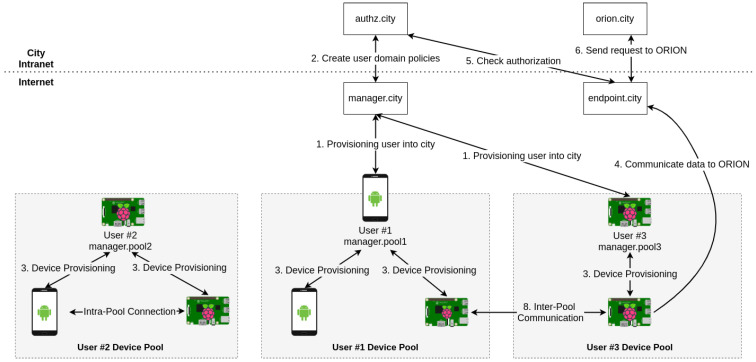
Setup integration with FIWARE.

In brief, the different steps of this setup are described in Figure 10, and are the following:

1. The user requests authentication with the city manager;
2. The city manager creates user domain policies;
3. Users can authenticate new devices on their pools. As the users are authenticated with the city, their devices are intrinsically authenticated with the city too;
4. An authenticated device tries to communicate data with Orion, sending the request to the endpoint of the city;
5. The endpoint receives the request and checks the authorization on AuthzForce component;
6. The AuthzForce answers with the authorization granted, and the endpoint forwards the device’s request to Orion.

Figure 10 also shows that it is possible to generate a device pool not connected to the city. These device pools are isolated and cannot access city services nor communicate with other nodes. To access city services, the pool needed to be appended to the city using manager provisioning. It is possible to communicate directly between nodes on other pools when both pools are appended to the PKI (both managers authenticate between them).

## 6. Smart Cities FIWARE Deployment

This section describes the integration between YubiAuthIoT and the FIWARE platform. We describe the configuration that mimics a smart city environment with devices representing managers and sensors in this integration.

### 6.1. Setup

The current implementation was tested by having four distinct devices under the same network and two YubiKeys, using the following configuration:

- Device #1:
  Device Info: OrangePi PC arm32 running armbian
  Components: CityBootstrapper, OTP Server (that can only verify the first Yubikey), authzforce-ce-server
  IP: 192.168.1.250
  USSN: Manager.city
  /etc/hosts: Unaltered
- Device #2:
  Device Info: Acer Nitro 5 amd64 running Ubuntu 20.10
  Components: EndpointBootstrapper, orion backend
  IP: 192.168.1.8
  USSN: Endpoint1.city
  /etc/hosts: 192.168.1.250 Manager.city
- Device #3:
  Device Info: Raspberry Pi 4 arm64 running Ubuntu Server 20.10
  Components: UserBootstrapper, OTP Server (that can only verify the second Yubikey)
  IP: 192.168.1.240
  USSN: Manager.pool1
  /etc/hosts: 192.168.1.250 Manager.city
- Device #4:
  Device Info: OrangePi Zero arm32 running armbian
  Components: DeviceBootstrapper
  IP: 192.168.1.150
  USSN: iotDevice.pool1
  /etc/hosts: 192.168.1.240 Manager.pool1, 192.168.1.8 Endpoint1.city

The hosts file needed to be changed for the USSN names to be converted into IP addresses to facilitate the node discovery. The OTP Server is a service coupled with a manager device that verifies the received OTP’s authenticity.

### 6.2. Implementation

A smart city deployment involves a series of components that must be deployed to achieve the desired functionality and security. Together with the launching of FIWARE Orion and FIWARE Authzforce, the following components must be launched:

- auth.bootstrappers.EndpointBootstrapper A client that connects to Orion and AuthzForce instance and can receive, check authorization, and answer Orion requests;
- auth.bootstrappers.CityBootstrapper Builds the city root, this element is responsible for appending to its tree Endpoints and Users;
- auth.bootstrappers.UserBootstrapper A manager that creates its device pool and then appends its pool to the city, enabling communication between both pools without losing administrative properties on the pool created (This manager can register new nodes independently of the other manager);
- auth.bootstrappers.DeviceBootstrapper Represents the construction of a normal device that belongs to a user that generates data (i.e., Android device capturing data from GPS).

The FIWARE components, together with the Endpoints and the City Manager, must be part of an intranet isolated from the outside world to protect unauthorized access to FIWARE Orion and FIWARE Authzforce. Endpoints and the City Manager are the only bridge from the outside world to the intranet, and their job is to provide secure and authenticated access to the intranet infrastructure.

The connection to Authzforce and Orion is done using HTTPS to ensure that requests for these services made by Endpoints or City Manager are reaching the desired component and that the confidentiality and integrity of communication with these components are guaranteed.

Now we will discuss the implementation of the several components, especially for the deployment of FIWARE and YubiAuthIoT at its core. Regarding the communication, we used GRPC instead of regular HTTP with JSON because GRPC reduces the amount of data transmitted over the network thanks to its proto buffers. JSON produces large, human-readable outputs, while proto buffers produce a compact binary output. GRPC requires the set of proto files (files ended with .proto) to be defined; a proto file contains the format of the messages sent and received by the node. In this implementation, every node that uses YubiAuthIoT to be provisioned and communicate as the same set of proto files produces consistency even when dealing with different operative systems or programming languages. We tested the implementation of the DeviceBootstrapper and UserBootstrapper on low power devices like Raspberry Pi’s and also high mobility devices, such as Android smartphones.

#### 6.2.1. Communication between Nodes

After the provisioning process, when an IoT device wishes to communicate to Orion, it needs to communicate with a city endpoint device using a confidential, authentic and integral communication channel. However, before the communication occurs, the IoT device needs to encode the HTTP request that Orion supports into something with the desired format to be sent through a GRPC enabled communication channel. One solution is to encode the entire HTTP request into a byte array. However, this solution consumes unnecessary network resources, and it is possible to improve it to fit into a smaller footprint by removing HTTP request headers and protocol demands, as in “GET/v2/entity…HTTP/1.1” can be compressed by removing the “HTTP/1.1” part of the header, removing unnecessary headers. Thus, computational stresses on the IoT resulting from encryption and digital signature procedures and the consumption of network resources are lower because a smaller packet is sent.

The communication between devices can be subdivided into authentication and secure channel formation protocols. When the authentication ends, both devices have guarantees about the identity of the communicating peers caused by the presence of certificates signed by some entity that both devices trust, together with digital signatures of messages also enforcing the origin of the received contents. It reduces the possibility of MitM attacks attempting to capture/alter/destroy messages mid-communication to defeat the authentication protocol.

#### 6.2.2. Connection with AuthzForce

After successful provisioning on the city network, IoT devices can send a request to FIWARE Orion. The request must be authenticated, and an authorization process must act before the message is forwarded to Orion.

Endpoint seats in our architecture as PEP, acting as a proxy between the IoT device and Orion. The PEP is responsible for integrating with the Identity Management and Policy Decision services to allow or deny the referral to Orion.

First, the IoT device must communicate with the Endpoint using the protocol described above. The communication protocol will generate an authentic, confidential, and integral channel that the IoT device can use to send requests that will be forwarded to Orion by the Endpoint. Upon receiving a request, the Endpoint will use the information of the IoT device certificate to check the identity of the IoT device and obtain the pool where the IoT device is provisioned. Having that information and together with the metadata of the Orion request sent through the YubiAuthIoT enabled secure channel (metadata includes the HTTP method, HTTP headers, and the HTTP message body), it sends that information over HTTP in the XACML format that matches the policy set for that pool. The policies for that pool are generated when the pool manager gets provisioned into the city infrastructure using the provisioning protocol discussed above. In the current implementation, the XACML policy created only allows IoT devices inside the same pool to create/update/delete entities on that pool; otherwise, an IoT device from another pool will be unauthorized to access data from other pools. The defined XACML policy also introduces rules to the subscription of entities. An IoT device can only be subscribed to updates on entities that belong to the same pool. An IoT device can produce several entities and perform multiple subscriptions. When the Authzforce component receives a request to verify the access to an entity by an IoT on a certain domain, it first gets all the active policies for that domain and then, using the information on the received XACML body, obtains the policy results. Policy decisions can be set to return “Allow” if all the policy decisions are “Allow” or to return “Allow” when only some of the policies return “Allow” but, in the current implementation, we choose to return “Allow” when all active policies return “Allow”.

#### 6.2.3. Connection with Orion

Having a favorable result from the Authzforce policy decision, the next step is for the Endpoint to forward the communication to FIWARE Orion. The received request must be decoded and used to generate a valid HTTP request, placing the method, URL, and body in the correct place on the HTTP request. The next step is to send the request to Orion and wait for a response. Upon receiving a response, it is encoded and compressed using the methods described above, where unnecessary headers and HTTP response components are removed to reduce the network resource consumption. The compressed and encoded response is then encrypted using AES with the shared symmetric key K produced on the authentication protocol of the communication, the encrypted result is signed with the Endpoint ECDSA private key, and the signed result is sent to the IoT device, completing the communication.

## 7. Security Analysis

In this section, we define a security analysis composed of two main components: a threat model that help other researchers to understand the threats associated with out implementations and the respective mitigation, from the defender’s perspective. Then, we define the attack-scenarios from an attacker’s perspective.

### 7.1. Threat Model

This section identifies the system (assets) and potential threats against the proposed system from the defender’s perspective.

#### 7.1.1. Physical Devices

The only SPOF in the manager is the OTP Server. However, this service can be switched off whenever it is not necessary to provision new devices. The manager device acts as a central validation point in the devices provisioning phase.

The manager device must be considered safe and reliable, and it must be guaranteed that the device will not be broken or stolen because it is the device that will authenticate the new sensors that enter the network.

If an attacker stoles the security token, it is possible to revoke the OTP Server’s secure token. Furthermore, it is possible to set an expiration time. The private key never leaves the YubiKey and can only be used by the owner that knows the PIN. Even if the YubiKey is lost or stolen, the PIN has three attempts, and, if failed, it requests a PIN Unlock Key (PUK) that also has three attempts. If all fails, the user must reset the YubiKey, which erases all the content from the YubiKey. For this reason, owners’ should also make a backup of the information contained on the YubiKey.

On the other hand, using a secure token helps protect against hacking, as physical access to the secure token is required to generate OTP.

#### 7.1.2. Configurations for Mitigation of Attacks

In the secure token, the PIN has three attempts, and, if failed, it requests a PUK that also has three attempts. If all fails, the user must reset the YubiKey, which erases all the content from the YubiKey. For this reason, owners’ should also make a backup of the information contained on the YubiKey.

#### 7.1.3. Device Surveillance and Revocation

When a device enters a pool, there is no more surveillance if the node is malicious or misbehaving. For this reason, the owner must make that process manually by inspecting possible misbehaving of nodes and revoke it if necessary.

### 7.2. Attack Scenarios

This section presents some attacks that are usually issues related to IoT solutions. We want to classify the protocol security based on a set of theorems, adapting those used by Afifi et al. [23] or Sousa, Patrícia R., et al. [50] to prove that an authentication protocol is secure.

There are some pre-defined protections in the proposed system, such as the use of a secure token that helps in protection against hackers, as it requires physical access to the secure token to generate the OTP.

#### 7.2.1. Security against Tag Impersonation Attacks

The secure token generates OTPs and stores the public key of the manager to be transmitted to trusted devices, which prevents the manager from being impersonated because even if the attacker can change its name, the attacker needs to have their data (authenticated OTP and its public key) in the secure token, which becomes impractical. The use of a secure token helps protect against hackers, as physical access to the secure token is necessary to generate the OTP.

#### 7.2.2. Security against Replay Attacks

A replay attack occurs when an attacker copies a message stream between two parties and repeats the flow of one or more parties. The secure token (for example, YubiKey) uses a set of volatile and non-volatile counters that guarantee that an OTP can no longer be used after being validated once [51].

#### 7.2.3. Man-in-the-Middle

MitM is an attack in which data exchanged between two parties is intercepted, logged in, or possibly altered by an attacker without the victim’s noticing. This type of attack can be a passive or active attack.

This system uses a secure token to generate the OTP and store the public key of the manager. An attacker cannot override the legitimate public key with an attacker’s public key because it is transmitted by the secure token (that generates the OTP that needs to be valid when verified by the OTP Server). Furthermore, internal nodes in the network need to be authenticated to enter into the trusted device pool. If CA is the third party that is compromised, the signed certificate is likely to be useless, as no one will trust it. If a MitM captures the CSR, it cannot do anything with it, as the only available key is the public key.

#### 7.2.4. Resistance to SPOF

A SPOF represents a central location in a computer system, which, if attacked or fails, causes the system to fail. For example, in PKI implementations, the certification authority represents a SPOF, as once it is compromised, all peers are also compromised. A SPOF is better for hackers because if hackers compromise that point, they compromise the whole system.

The use of certificates on YubiKeys aimed to not expose private keys on centralized online devices. In other words, having a solution where keys that authenticate devices are stored and can make the cryptographic operations offline limits the exposure of those keys to malicious users.

Concerning databases, each one represents a SPOF. However, as there are several devices/managers in different departments, the system limits the centralization. Even in the home context, each home has its data, unlike a central database server.

When the system is in the provisioning phase, it has a SPOF in the manager that is authenticating the devices. This system employs a hybrid model to mitigate this problem, where the manager must be online only when needed and can be switched off when there are no new peers to connect to the network. In this situation, the system proposed in this paper is a decentralized model of communication between different things.

## 8. Future Research Challenges

This section describes future research challenges that can complement this work. In the current implementation, the manager’s identity is associated with the manager device’s identity, which means that the manager does not have a (human) identity. However, it might be interesting to authenticate these users with *eIDAS* and associate their authenticated devices with their human identity. The advantage of having the manager authenticated with its (government) identity is that users are guaranteed to be trusted peers that will not change the identity characteristics of the device. Álvaro Alonso et al. [52] propose a model that enables the connection of FIWARE OAuth 2.0-based services with the electronic IDentity (eID) authentication provided by *eIDAS* (eIDAS (electronic IDentification, Authentication and trust Services) is an EU regulation on electronic identification and trust services for electronic transactions in the European Single Market.) reference. With this, services already connected with an OAuth 2.0 identity provider can be automatically connected with *eIDAS* nodes for providing eID authentication to European citizens.

To empower the data minimization, the Idemix [53] concept can be integrated to have partial identities for devices, as in some cases, it may not always be necessary to send all the details of the identity, but instead, a proof that has something. Through protocols and cryptographic mechanisms, the schemes implemented by each model allow the presentation of credential authentication through credentials and proofs of attributes, preserving anonymity.

As a limitation that we have shown in the threat model is the lack of surveillance of network nodes after being provisioned, it is necessary that, in addition to the revocation that is already possible, automation of node surveillance can be implemented in the future, for example, possibly through a reputation system [54]. This solution can also help to mitigate SPOF in the manager. This reputation system has the agreement and participation of all nodes, where there would be compensation in case of good behavior and positive interactions of approval of nodes in the network, but also a penalty system (which would be even stronger than the system of compensation) that would deduct points from malicious nodes and potentially put them outside the network or in a sort of quarantine.

Furthermore, for devices misbehaving, it is interesting to consider technologies such as Certificate Transparency [55,56], which is an ecosystem that makes the issuance of website certificates transparent and verifiable. Google Trillian [57] is an example that helps to detect misbehavior of CAs by enforcing that all certificates are in a verifiable log.

Alternatives to *zeroconf* and *mDNS* for network discovery can also be implemented and developed. Despite limitations such as IoT not having a screen, an IP could be shown—in a QR-code, for example—on a target device that can be read or inserted into the controller, and thus we can overcome the problems of discovery technologies.

## 9. Conclusions

With this solution, we have several benefits on authentication of IoT devices: device identity, scalability, offline cryptographic assets, revocation and dissemination, and resistance to MiTM. It is possible to have device pools, which in addition to authenticating multiple devices, can authenticate with other device pools (for example, different entities or departments), allowing us to scale the solution to provision and authenticate multiple devices.

YubiAuthIoT allows a more decentralized provisioning and device management, recurring to subCAs and pools, reducing the need for a discrete IdM service to manage devices. YubiAuthIoT provides a more efficient and more resilient authentication and communication than the default in the FIWARE platform, which is based on HTTPS over HTTP/1.1 with JSON bodies, where the authentication is provided by access tokens requested by each device/user and provided by the centralized IdM service. The usage of certificates introduces the same capabilities as access tokens while not requiring an IdM centralized service to be deployed, which creates the possibility of SPOF attacks that, since the IdM is the core component of token generation and verification, break the systems entirely.

Having the private keys and the associated public-key certificate on a hardware token reduces the possibility of attacks that attempt to extract the private key from the device. In other words, having a solution where the keys that authenticate the devices are offline limits the exposure of these keys to malicious users. The usage of OTPs produced by the same hardware token enables the manager devices to have guarantees that the correct device owns the hardware token.

Often, the reluctance to use these types of solutions is based on smart cards, which require external readers and computer libraries. However, this work uses a secure token that only requires a USB port, and given the study by authors Kiran Jot Singh and Divneet Singh Kapoor [58], there are USB ports on most devices used in IoT.

In the absence of a USB, it is possible to adapt this system with NFC as the Yubikey features this technology. Of course, this type of provisioning requires modifying the software at manufacturing time, but it is a security-by-default mechanism necessary to employ in IoT devices. Then, it is also necessary to provision and authenticates the devices to each other before being used. With the secure token, it is possible to provision a device to an existing network in production mode.

Additionally, YubiAuthIoT can be easily integrated with components like FIWARE IoT agents, providing authenticity and secure channels, not reducing the usability of already defined FIWARE components.
